# Severe hemolysis with negative direct antiglobulin test: A case report

**DOI:** 10.1016/j.amsu.2022.104444

**Published:** 2022-08-18

**Authors:** Mahin Behzadifard, Ali Arianezhad, Ali Bandehzadeh, Mohammadali Gholampour

**Affiliations:** a Dezful University of Medical Sciences, Dezful, Iran; b Department of Medicine, Lung Biology Center, Cardiovascular Research Institute, University of California, San Francisco, CA, USA

**Keywords:** Autoimmune hemolytic anemia, Anti-human globulin, Coomb's test, DAT, Direct antiglobulin test

## Abstract

A 49-year-old woman with type 2 diabetes mellitus (T2DM) presented to the emergency department. Her examination showed marked pallor, exhaustion, lethargy, yellowish eyes, anorexia, nausea and vomiting.

Hematuria; negative standard direct antiglobulin test (DAT); normal glucose 6 phosphate dehydrogenase (G6PD); hemoglobin (Hb), 4.8 g/dl; Mean cell volume (MCV), 91fl; platelet count, 233 × 10^6^/L; Total bilirubin, 7.0 mg/dl; Glucose, 316 mg/dl; lactate dehydrogenase (LDH), 1750U/L. Undoubtedly, therapeutic panel should have been used for hemolytic anemia. Intravenous (IV) fluids and 2 units of packed cell were transfused. Methylprednisolone with rituximab were started for the patient. After 3 weeks of the patient admission, she was discharged home with stable vital signs and Hb, 10 g/dl. We concluded in the cases that presented along with a severe drop in Hb and evidence of hemolysis which non immune hemolytic anemia is excluded in spite of negative standard DAT limited transfusion besides corticosteroids combined with rituximab, could be helpful in saving the patient.

## Highlights

• Autoimmune hemolytic anemia (AIHA) is caused by increased erythrocyte destruction with autoantibodies directed against the person's own red blood cells and susceptible them to lyse and consequent insufficient number of red blood cells in the circulation.
• A diagnosis of DAT-negative AIHA can be made following careful exclusion of alternative causes of hemolysis, and confirmation by a sensitive technique and by a response to steroid therapy.

## 1 Introduction and importance

**Fig. 1:**
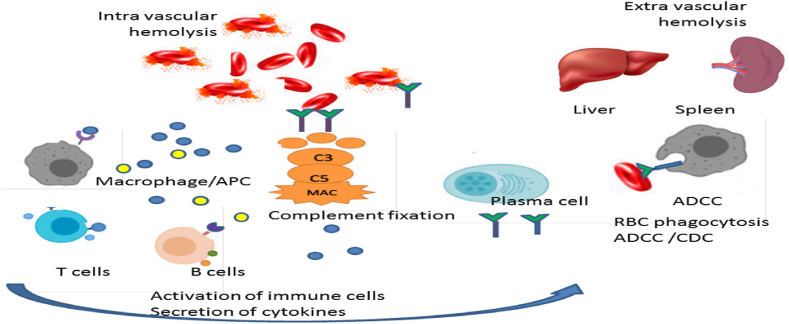
The various immunologic mechanisms involved in AIHA pathogenesis, including macrophages, APC, B and T lymphocytes, cytokines, complement cascade, ADCC in the spleen, and/or CDC in the liver. MAC, membrane attack complex; ADCC, Antibody-dependent cell mediated cytotoxicity; APC, antigen presenting cells; CDC, complement-dependent cytotoxicity.

Hemolytic anemia can be divided into immune and nonimmune types. Autoimmune hemolytic anemia (AIHA) is caused by increased erythrocyte destruction with autoantibodies directed against the person's own red blood cells and susceptible them to lyse and consequent insufficient number of red blood cells in the circulation. The disease may be primary, or secondary to an underlying illness such as drug induced, associated with lymphoproliferative neoplasms, solid tumors, or transplants, autoimmune and infectious diseases and immunodeficiencies [[1], [2], [3]]. The primary form is idiopathic and accounts for approximately 50% of cases [4,5]. Warm or cold antibody is responsible in AIHAs. Warm AIHA (wAIHA) as the most common type of AIHA is associated to immunoglobulin G (IgG) autoantibodies (60–70% of all cases). In the present at high titer IgG1 and IgG3 subclasses complement activation occur, but other types of IgG typically can't fix complement, therefore induce extravascular hemolysis. Extravascular hemolysis occurs in antibody-dependent cell mediated cytotoxicity (ADCC) by macrophages and activated lymphocytes in the lymphoid organs and spleen [6]. Multiple immunologic mechanisms involvement that have variable role over the time make wAIHA as a very heterogeneous disease with an unpredictable clinical course (Fig. 1) [7,8].

The AIHA diagnosis can be made with a step by step approach that used the laboratory and clinical evidence and determined the immune nature of hemolysis with the direct anti-globulin test (DAT) that is positive in 70% of all wAIHA or anti-IgG plus complement at low titer [5]. Occasionally wAIHA can have IgM or IgA nature. Cold AIHA is typically caused by IgM antibodies that bind and agglutinate RBCs at low temperatures and after complement fixation intravascular hemolysis occurred [9].

Identifying hemolytic anemia with a positive DAT is made by clinical assessment of obvious causes such as delayed transfusion reaction subsequent to a recent transfusion, alloimmune hemolytic anemia following allogeneic stem cell or solid organ transplantation, drug-induced immune hemolysis, or hemolytic disease of the fetal or newborn. In 3–11% of AIHA cases DAT is negative by standard protocol. A diagnosis of DAT-negative AIHA can be made following careful exclusion of alternative causes of hemolysis, and confirmation by a sensitive technique and/or by a response to steroid therapy [10,11]. DAT may be falsely positive in healthy individuals, after therapies such as IVIG, Rh immune globulins, anti-thymocyte globulins, daratumumab, in recently transfusion (alloantibodies), in paraproteinemia or elevated serum globulins, in patients with AIHA in remission, so that AIHA cannot be diagnosed from positive DAT alone and it's necessary to search hemolysis evidence, and other congenital or acquired hemolytic disorders should be excluded in complex cases [12,13]. Therapeutic interventions include steroids and splenectomy or rituximab as the first and second-line treatments or immunosuppressants. Recently rituximab is increasingly used in steroid-refractory cases based and experimental therapies that directed at complement such as proteasomes, and different kinases are under development [14,15]. In this report our aim was to present a case that in spite of the patient DAT and G6PD were respectively negative and normal but other laboratory investigations such as peripheral blood containing microspherocyte, elevation of bilirubin and LDH emphasis to the presence of a hemolytic anemia. So that limited transfusion and by considering of sever decreased in Hb corticosteroid and rituximab were started for the patients and after 3 weeks the patient with Hb: 10g/dl was discharged.

### 1.1 Case presentation

The work has been reported in line with the SCARE 2020 criteria [16]. The patient data of a 49-year-old woman originated from south west of Iran that was admitted to the emergency department mid-august 2020 for marked pallor, exhaustion, lethargy, yellowish eyes, anorexia, nausea and vomiting was collected. Past medical history revealed T2DM for over 10 years, hyperlipidemia and drug consumption of metformin 500, atorvastatin 20, pioglitazone 30, ASA 80, and Cetirizine. Laboratory analysis on admission showed; Hb, 4.8 g/dl; MCV, 91fl; platelet,233 × 10^6^/μl; WBC,9.8 × 10^6^/L; total bilirubin, 7.0 mg/dl; glucose, 316 mg/dl; Ferritin, 1490 ng/ml; G6PD, normal; direct coomb's, negative; hematuria; and urine sugar, +3. The peripheral blood smear showed some spherocyte and micro-spherocyte and no schistocytes; 4% immature granulocytes and 3% nucleated red blood cells (NRBCs). The serum haptoglobin level was not determined. Due to clinical and laboratory evidence of hemolysis, transfusion and hydration were performed. Serum normal saline and severe diuresis in the range of 1.5–2 cc/kg/h with furosemide were used for the patient. In spite of receiving 2 units of packed cells and methylprednisolone (1.5mg/kg per day), in the first 3 days of hospitalization, on day 4 the patient's condition worsened and the patient's Hb was dramatically decreased with Hb level 4.3g/dl (Table 1) and she was transferred to ICU. O- negative pack cell with corton pulse (1 g hydrocortisone) and chlorpheniramine were slowly infused, Rituximab, and due to the high risk of the patient in terms of deep vein thrombosis (DVT) and pulmonary embolism (PE), prophylaxis with enoxaparin was started. At the 5th day of hospitalization, the patient lost her consciousness and she was intubated, she suffered cardiac arrest during the intubation process, which was revived after 15 minutes of cardiopulmonary recovery. Gradually, the patient's level of consciousness returned to normal her general condition was improving and he had regained consciousness and treatment with limited transfusion, corton pulse, chlorpheniramine, methylprednisolone and rituximab was continued. After one week the patient's Hb reached near to 7 g/dl, but unfortunately on the same day despite a good response she suffered from sudden chest pain. We previously started prophylaxis of enoxaparin for the patient, but the symptoms of PE were obvious. Because the patient was hospitalized in the ICU and CT angiography was not possible but clinical signs, D-Dimer test and portable radiography confirmed the diagnosis of PE. Enoxaparin was converted from a prophylactic dose to a therapeutic dose. On the 9 day of hospitalization, the patient was in good general condition and her vital signs were stable and the patient was cared for 1 week later in general ward. After 3 weeks of patient admission, she was discharged home with stable vital signs and Hb10 g/dl.

**Table 1:** Laboratory data on the first 7 days of admission.

Indices	Day 1	Day 2	Day3	Day4	Day5	Day6	Day 7
Red blood cells ( × 10^12^/L)	1.4	1.9	1.59	1.1	1.5	1.6	2.02
Hb (g/dl)	4.8	6.0	5.3	4.3	5.7	5.0	6.5
Total bilirubin (mg/dl)	7.0	6.4	5.8	4.2	8.1	8.3	9.7
LDH (U/L)	1750	1810	1890	1907	1441	1242	1337
Hematocrit (%)	14.6	17.8	15.9	11.2	13.1	14.3	18.4
Platelets ( × 10^6^/L)	233	166	207	145	183	141	195

## 2 Discussion

When a patient presents with anemia; a stepwise approach is followed. The diagnosis of AIHA can be made with laboratory and clinical evidence of hemolysis and then determine the immune nature of hemolysis with DAT. Decreased RBC count, Hb and Hematocrit normo-macrocytic anemia, increased reticulocyte count, raised indirect bilirubin and LDH, reduced serum haptoglobin, and blood smear features with polychromasia or spherocytes, schistocyte and agglutination may be helpful for the physician to diagnosis of hemolysis as the cause of the anemia [5,17]. In this patient's medical history showed no hemolytic risk factors and her blood G6PD test was reported normal. Laboratory findings are shown in this case report during the first 7 days of admission (Table 1). Increased serum LDH and dropped Hb consistently in spite of transfusion confirmed hemolytic anemia, but patients with AIHA should be having restrictive transfusion and do not liberal transfusion because it can lead to hyperhemolysis. In this patient, clinical evidence and laboratory findings indicate hemolytic anemia but DAT test was negative. Additionally, platelet count was normal and the peripheral blood smear on admission showed anisocytosis, normo-macrocytosis, polychromasia, some spherocytes and micro-spherocytes and no schistocytes that confirm extravascular immune hemolytic anemia. Our work up has limitation such as no assessment of serum haptoglobin level and no super coombs testing evaluation. Immune mediated hemolysis or G6PD deficiency have been known as two main causes of hemolytic anemia [18,19]. The precise incidence of presentation of patients that have an anemia compatible to wAIHA and a negative DAT has been estimated at 3–11% of all cases [[20], [21], [22]]. Different causes for this finding included possible hemolysis by natural killer cells (NK cells) independent of antibody, presence of low affinity IgG that removed by preparatory washes protocols, sensitization below the threshold of detection of the commercial antiglobulin reagent (anti-human reagent potency), IgA or IgM autoantibodies, red cell sensitization by IgA alone, or rarely monomeric-IgM alone, that not accompanied by complement fixation, and therefore not detectable by a commercial polyclonal antiglobulin reagent. Due to these different possibilities, a negative DAT must be interpreted in conjunction with clinical and other laboratory findings. If clinical suspicion is high and research into non-immune causes is not justified, in addition more sensitive than the standard DAT protocol including microcolumn, solid phase, washings with cold or low-ionic salt solutions that may be helpful, the treatment for AIHA should be started [12,19,[23], [24], [25]]. Various pathogenetic mechanisms may responsible in AIHA so that different therapies administered. Steroids as first line and rituximab as the second line treatments to decrease inﬂammation and the B lymphocytes that produce antibody, IVIg to mask ADCC, PEX to remove excess immune mediators, and recombinant erythropoietin to induce increase in erytheropoiesis. Rituximab is usually recommended as a second-line therapy; however, in urgent situations, the exact time of steroid failure is not easy to deﬁne. In AIHAs such as following hematopoietic stem cell transplantation that is generally severe, refractory to steroids, and fatal, rituximab is recommended either as a frontline or early second-line therapy [3,26,27].

## 3 Conclusion

In AIHA that presented with negative standard DAT and severe drop in Hb that non immune hemolytic anemia is excluded and clinical and paraclinical findings confirm the presence of hemolysis, pay special attention to immune mediated hemolysis and do not be misled by a negative coombs test. In this situation limited transfusion besides corticosteroids accompanied with rituximab therapy could be helpful in saving the patient.

## Ethical approval

This case report approved code of ethics (IR.DUMS.REC.1401.012) from Dezful University of medical sciences, Dezful. Iran.

## Sources of funding

Not applicable.

## Author contribution

All authors contribute designed the subject of paper, search data base and write the paper. The author(s) read and approved the final manuscript.

## Registration of research studies

1. Name of the registry:

2. Unique Identifying number or registration ID:

3. Hyperlink to your specific registration (must be publicly accessible and will be checked):

## Guarantor

Mahin Behzadifard.

Corresponding author.

## Consent

Written informed consent for participation of the case report was obtained from the patient.

## Declaration of competing interest

The authors declare that they have no competing interests.
